# Strong textured SmCo_5_ nanoflakes with ultrahigh coercivity prepared by multistep (three steps) surfactant-assisted ball milling

**DOI:** 10.1038/srep13117

**Published:** 2015-08-14

**Authors:** Wen-Liang Zuo, Xin Zhao, Jie-Fu Xiong, Ming Zhang, Tong-Yun Zhao, Feng-Xia Hu, Ji-Rong Sun, Bao-Gen Shen

**Affiliations:** 1 State Key Laboratory of Magnetism, Institute of Physics, Chinese Academy of Sciences, Beijing, 100190, People’s Republic of China

## Abstract

The high coercivity of 26.2 kOe for SmCo_5_ nanoflakes are obtained by multistep (three steps) surfactant-assisted ball milling. The magnetic properties, phase structure and morphology are studied by VSM, XRD and SEM, respectively. The results demonstrate that the three step ball-milling can keep more complete crystallinity (relatively less defects) during the process of milling compared with one step high energy ball-milling, which enhances the texture degree and coercivity. In addition, the mechanism of coercivity are also studied by the temperature dependence of demagnetization curves for aligned SmCo_5_ nanoflakes/resin composite, the result indicates that the magnetization reversal could be controlled by co-existed mechanisms of pinning and nucleation.

Nanostructured Co-based rare earth permanent magnetic compounds with high coercivity and strong texture have drawn much attention due to their high temperature application and high performance soft/hard exchange coupled magnets^1^,^2^,^3^. Lately, the surfactant-assisted ball milling (SABM) method has been found efficient in the textured nanostructured rare earth compound synthesis^4^. However, the texture degree and coercivity still have much room for improvement. It is known that most of the magnetic materials are brittle when the size of the particles is large, and with the particle size decrease, the brittleness decrease and ductility increase. It is necessary to explore a higher energy to crush the sample into a small size^5^,^6^. Therefore, high-energy ball milling (BM) method is often used in the process for preparing the SmCo_5_ nanoflakes^6^,^7^,^8^,^9^,^10^,^11^,^12^, However, the high energy during the BM process may more easily destroy the crystal structure. In order to decrease the defects from BM, low-energy BM experiments were carried out for preparation nanostructured magnetic materials, and obtained excellent magnetic properties^13^,^14^. However, for some hard materials, low-energy is hard to crush the sample into a smaller size especially for a nano-scaled size. Therefore, a connected method of multistep (three steps) BM method is used to solve those questions. In this paper, we adopt a low-energy in the initial stage of BM, and with the decrease of powder size, the energy are concertedly increased. In the end, we find that it is a good method for preventing the destruction of crystal structure and obtaining the strong textured nanoflakes with high coercivity.

## Results and Discussion

**Figure 1:**
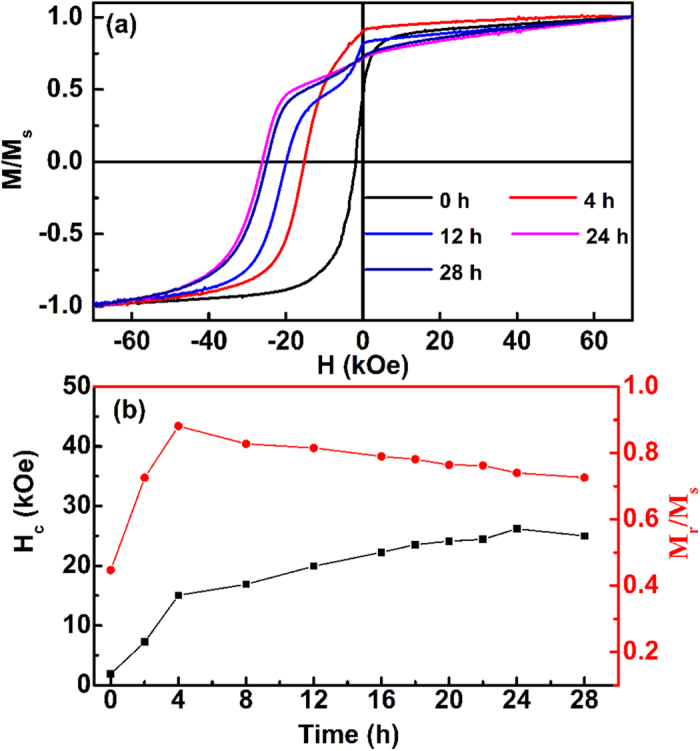
(**a**) The demagnetization curves of SmCo_5_ powders with the BM time from 0 to 32 h. (**b**) The coercivity *H*_c_ and remanence ratio *M*_r_/*M*_s_ dependence on BM time.

Figure 1(a) shows the demagnetization curves of SmCo_5_ powders with the BM time from 0 to 32 h. The coercivity *H*_c_ and remanence ratio *M*_r_/*M*_s_ dependence on BM time are also shown in the Fig. 1(b). It can be seen that both the *H*_c_ and *M*_r_/*M*_s_ show a sharp increase in the initial stage of BM (time ≤ 4 h), which is mainly attributed to the grain refinement. This result demonstrates that the low-energy (30 V, 150 rpm) is effective in the grain refinement especially for the initial stage of BM. With increasing the BM time and energy, *H*_c_ only shows a smooth increase and reaches a maximum value of 26.2 kOe after 24 h, which is the maximum value of coercivity for the reported rare earth permanent magnetic nanoflakes, and even higher than Tb-Fe-B^15^. Meanwhile, the decline in increase rate of coercivity can be due to the brittleness decreases and ductility increases, which lead to the decrease of grain refinement efficiency when the particle size decreases into nano-scaled^5^,^6^. In the end, the slight decrease of coercivity for BM 28 h indicates that the BM energy could not need to further improve. To the contrary, the *M*_r_/*M*_s_ shows a monotonous decrease when the BM time is larger than 4 h, this phenomenon is normal and owing to multi-factor. Such as, the increase fraction of small polycrystalline nanoflakes and nanoparticles which incline to random orientation and incoherence in grain boundaries^10^, the decrease uniaxial (00*l*) texture due to the plastic deformation^11^. The decrease of *M*_r_/*M*_s_ also indicates that the high energy is harmful for forming strong textured nanoflakes. Therefore, in this paper, the maximum BM energy is no longer increase and fixed as 50 V (about 250 rpm).

**Figure 2:**
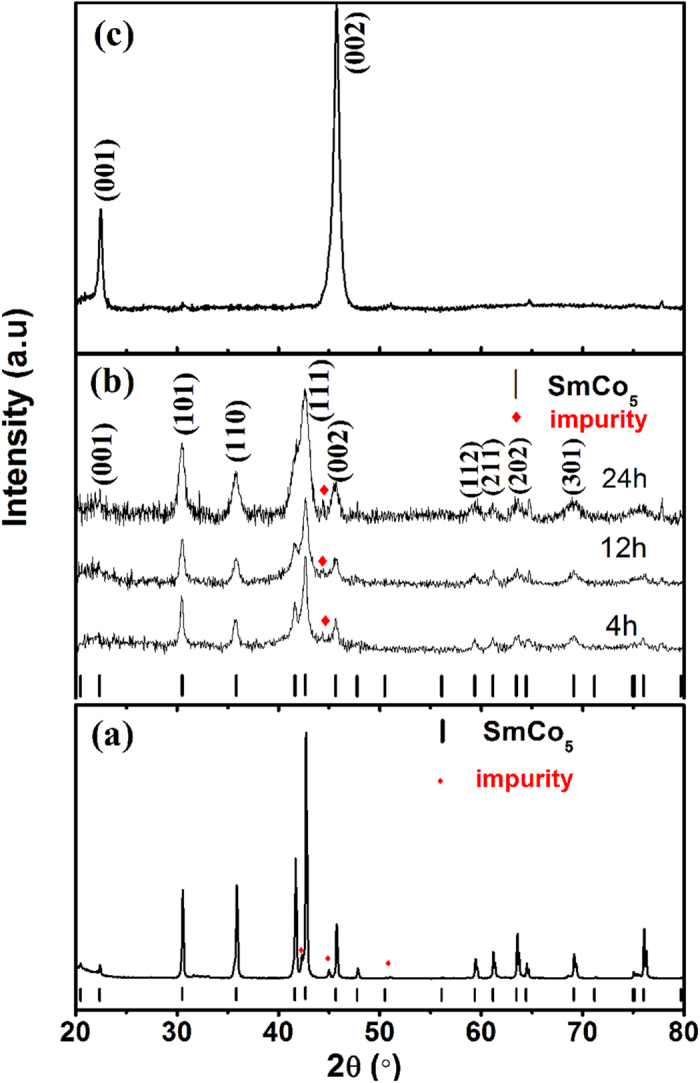
The XRD patterns of (**a**) starting SmCo_5_ compound powder, (**b**) as-milled SmCo_5_ powder with BM time from 4 to 24 h, (**c**) aligned sample of SmCo_5_ nanoflakes with BM time of 24 h.

Figure 2(a) shows the XRD pattern of starting SmCo_5_ compound powder, which crystallizes primarily in the hexagonal SmCo_5_ phase (JCPDS PD#65–4844) and with minor impurity. The XRD patterns of as-milled samples are shown in Fig. 2(b). It can be seen that the diffraction peaks become broader with increasing the BM time, which is due to the grain refinement and the introduction of the internal stress during the BM process. The average crystallite size calculated via Scherrer’s formula is approximately 20 nm, 12 nm, and 6 nm, and the internal strain is about 0.16%, 0.32%, and 0.31%, corresponding to the ball milling time of 4 h, 12 h, and 24 h, respectively. Because of the relatively low BM energy, especially for 4 h milling, the broadening diffraction peaks mainly come from grain refinement. Therefore, this broadening also demonstrates that the low BM energy (150 rpm) is effective in the grain refinement. Because of the low energy instead of high energy in the initial stage of BM, the defects of crystalline structure can be decreased in the whole BM process. In addition, the XRD pattern of aligned sample (milled for 24 h) is shown in Fig. 2(c). It can be seen that the diffraction intensity of (00*l*) crystalline planes dramatically enhances whereas that of the other peaks almost disappear, suggesting that a strong (00*l*) alignment is obtained for the aligned sample (the easy magnetization directions along the *c*-axis).

**Figure 3:**
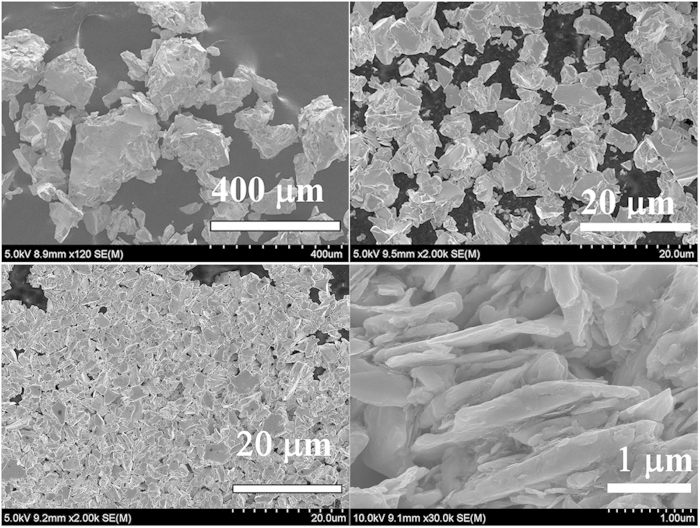
The SEM images of SmCo_5_ powder with different BM time.

Figure 3 shows the morphology evolution of nanoflakes with the BM time from 0 to 24 h. It is obviously that the start powder is irregular shape and the size are around 50–400 μm. After 4 h low energy BM (150 rpm), the start powders are crushed down to smaller particles and with the average diameter of 5 μm, which more intuitively demonstrates that the low energy is effective in the grain refinement. However, the morphology of sheet type can hardly be seen. With the increase of milling time and milling energy reach to 12 h and 200 rpm, respectively. The particles become smaller and more uniformly. Some sheet-type morphology, with micron or submicron thickness and 1–5 μm length, can be seen obviously. With further increasing milling time and energy, the SmCo_5_ nanoflakes, with thickness about 50–200 nm and length in the range of 1–2 μm, are prepared. Furthermore, the nanoflakes form “kebab-like” morphology due to the *c*-axis texture and magnetostatic interaction, which indicates that the easy magnetization direction of as-milled SmCo_5_ nanoflakes is perpendicular to the surface of the flakes. It is interesting that the nanoflakes with multistep BM shows smaller length and aspect ratio compared with those of usual one step high energy BM^6^,^7^,^8^,^9^,^10^,^11^,^12^, which is favorable for decreasing the demagnetization fields of SmCo_5_ nanoflakes.

**Figure 4:**
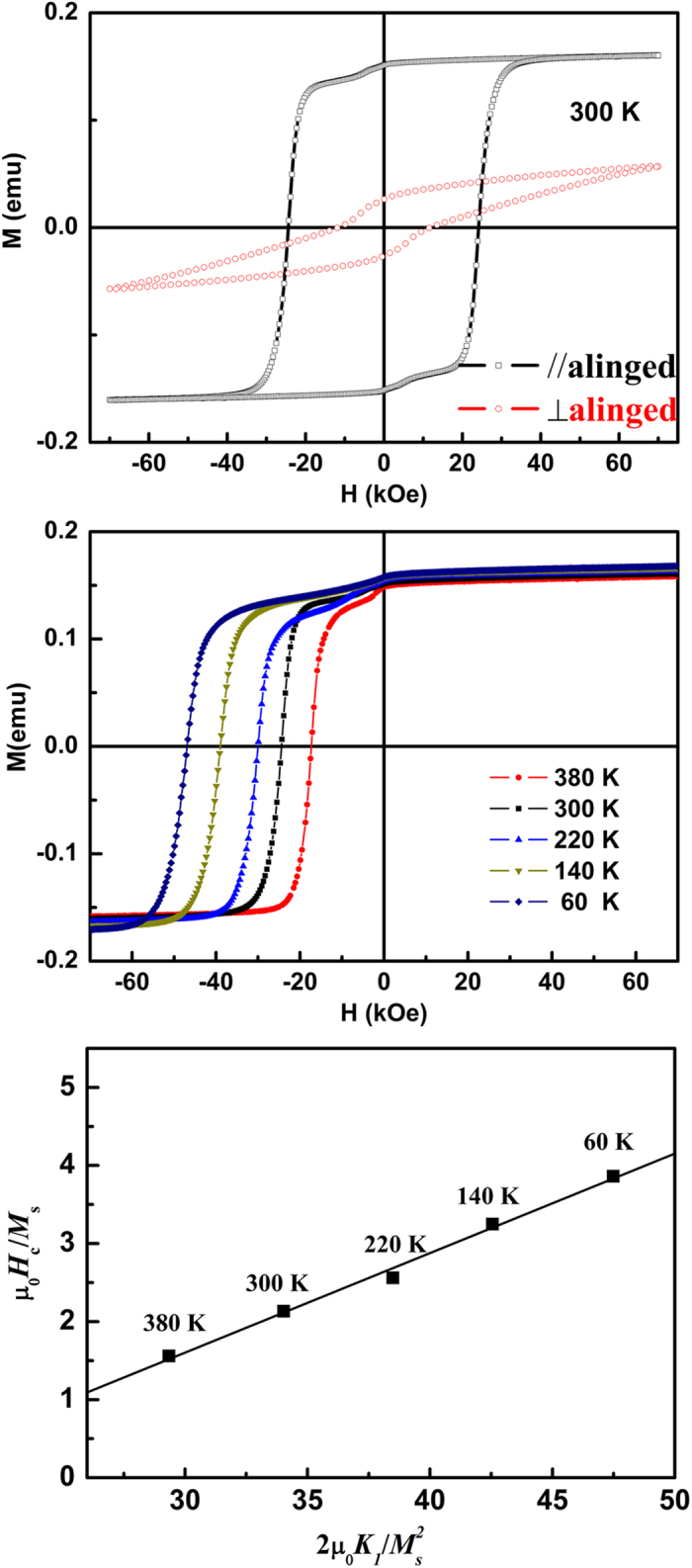
(**a**) The hysteresis loop, (**b**) temperature dependence of demagnetization curves and (**c**) 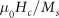 against 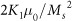
on different temperature for aligned SmCo_5_ nanoflakes/resin composites with by 24 h BM.

Figure 4(a) shows the hysteresis loops of aligned SmCo_5_ nanoflakes prepared by 24 h three steps SABM. The obvious anisotropy magnetic behaviors are observed. However, the *H*_c_ (24.4 kOe) for the aligned sample shows an obviously decrease compared with that of unaligned one (26.2 kOe) (see Fig. 1(a)), which phenomenon is also observed for many rare earth permanent magnetic materials^16^,^17^,^18^. In addition, the *M*_r_*/M*_s_ reaches 0.94 for parallel direction of the easy axis, which indicates that the sample have a good alignment degree. In order to more accurately describe the alignment degree, we also calculate the average misalignment angle, 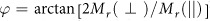
7,19, where *M*_r_(┴) and *M*_r_(||) are the remanence of perpendicular and parallel direction of the easy axis, respectively. The misalignment angle φ = 19°, which is smaller than the experiment results of BM in the magnetic field^7^,^20^, indicates that the nanoflakes with three steps SABM have a higher texture degree.

In order to study the mechanism of coercivity for the SmCo_5_ nanoflakes, the temperature depen-dence of demagnetization curves for aligned SmCo_5_ nanoflakes/resin composite are shown in Fig. 4(b). According to the micro magnetic model, the coercivity can be generally expressed as^21^,^22^,^23^: 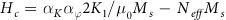, where *K*_1_, *N*_*eff*_, and *M*_s_ are the first-order anisotropy constant, the effective local demagnetization factor and the saturation magnetization, respectively. The coefficient α_K_ incorporates the effect of the sample microstructure, especially inhomogeneous of the intrinsic material parameters, and *α*_φ_ describes the information of the easy axis misaligned. These parameters can be determined by linear fitting 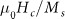 against 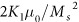 (See Fig. 4(b)). The temperature dependent values of *K*_1_ and *M*_s_ are taken from the measurements of SmCo_5_ single crystal^24^. The obtained α_K_*α*_φ_ and *N*_*eff*_ are 0.13 and 2.22, respectively. For the aligned samples, the α_K_*α*_φ_ value of 0.13 is almost entirely attributed to the microstructure parameter α_K_ due to the small misalignment angle, and this value is close to that of epitaxial SmCo_5_ thin films^22^, therefore, it indicate that the as-prepared nanoflakes have a similar microstructure (high textured but inhomogeneous nanocrystalline structure) compared with the epitaxial SmCo_5_ thin films. In addition, the α_K_*α*_φ_ value of 0.13 also indicates that the magnetization reversal may be controlled by both nucleation and pinning (α_K _< 0.3)^22^,^23^. The value of *N*_*eff*_ is larger than 1 and close to the ball milled MnBi magnets^13^, which indicates that large stray field could be existed in this sample except the effective local demagnetization factor. In the end, it is need to note that this paper only offer a relatively easy method (multistep BM) for preparation the nanoflakes with strong texture and high coercivity, however, it is not the optimal technological process for multistep BM. Therefore, it is need more detail experiments to develop this method.

The high coercivity of 26.2 kOe for SmCo_5_ nanoflakes are obtained by multistep (three steps) SABM, which is the maximum coercivity of reported nanoflakes permanent materials. All the results of XRD, VSM and SEM demonstrate that the low-energy (150 rpm) is effective in the grain refinement especially in the initial stage of ball milling, however, it is need to note that low-energy is only effective in the initial stage, the higher-energy is also need for the further crush. Compared with one-step high energy BM, the three steps BM can keep more complete crystallinity during the process of milling (relatively less defects), which enhance the texture degree and coercivity. The strong textured SmCo_5_ nanoflakes with high coercivity are very promising for future development of anisotropy nanocomposite magnets and high performance soft/hard exchange spring magnets.

## Methods

SmCo_5_ ingots were purchased from Taiyuan Tianhe Hi Tech Co Ltd. The as-obtained ingots were annealed at 1173 K for a week under vacuum, and then ground down to less than 400 μm as the starting powders. The BM experiment was performed for three steps using a GN-2 BM equipment: 4 h with the voltage of 30 V (speed is about 150 rpm); 8 h with the voltage of 40 V (speed is about 200 rpm); 16 h with the voltage of 50 V (speed is about 250 rpm). The weight ratio of balls to powders was 20:1. Oleylamine (80%–90%) and oleic acid (99%) were used as surfactants. The total amount of surfactants was 20% to the weight of the starting powders (Oleylamine and oleic acid was 1:1). Heptane (99%) was used as the carrier liquid. The aligned SmCo_5_ nanoflakes/resin composite was prepared by mixing the as-milled nanoflakes with epoxy resin, and placing them into a 20 kOe magnetic field until the epoxy resin solidifies. The phase structure was examined by the X-ray powder diffraction (XRD) with Cu Kα radiation at room temperature. Morphology was analyzed by scanning electron microscope (SEM). Magnetic properties were measured by a SQUID VSM with the maximum field of 70 kOe.

## Additional Information

**How to cite this article**: Zuo, W.-L. *et al.* Strong textured SmCo_5_ nanoflakes with ultrahigh coercivity prepared by multistep (three steps) surfactant-assisted ball milling. *Sci. Rep.* **5**, 13117; doi: 10.1038/srep13117 (2015).
